# *ADH1B* and *CDH1* polymorphisms predict prognosis in male patients with non-metastatic laryngeal cancer

**DOI:** 10.18632/oncotarget.12301

**Published:** 2016-09-28

**Authors:** Daxu Li, Ruizhi Zhang, Tianbo Jin, Na He, Le Ren, Zhe Zhang, Qingna Zhang, Ran Xu, Hong Tao, Guang Zeng, Jing Gao

**Affiliations:** ^1^ Department of Stomatology, First Affiliated Hospital, Xi'an Jiaotong University School of Medicine, Xi'an, Shaanxi 710061, China; ^2^ Department of Stomatology, Ankang Central Hospital, Ankang 725000, Shaanxi, China; ^3^ School of Medicine, Xizang Minzu University, Xianyang, Shaanxi 712082, China; ^4^ Department of Plastic and Burn Surgery, Tangdu Hospital, The Fourth Military Medical University, Xi'an, Shaanxi 710038, China; ^5^ State Key Laboratory of Military Stomatology & National Clinical Research Center for Oral Diseases & Shaanxi Key Laboratory of Stomatology, Department of Prosthodontics, School of Stomatology, The Fourth Military Medical University, Xi'an, Shaanxi 710032, China

**Keywords:** laryngeal cancer, polymorphism, prognosis, ADH1B, CDH1

## Abstract

In this study, we assessed the association between single nucleotide polymorphisms (SNPs) in candidate genes and the prognosis of laryngeal cancer (LC) patients. Thirty-seven SNPs in 26 genes were genotyped in 170 male Han Chinese patients with LC. The effects of the candidate genes on the prognosis of LC patients were evaluated using Kaplan-Meier curves and Cox proportional hazards regression models. The GA genotype of rs1229984 (hazard ratio [HR], 0.537; 95% confidence interval [CI], 0.340–0.848; *p* = 0.008) in *alcohol dehydrogenase 1B (ADH1B)*, and the AA genotype of rs9929218 (HR, 6.074; 95% CI, 1.426–25.870; *p* = 0.015) in *CDH1* were associated with overall survival. Our data suggest that polymorphisms in *ADH1B* and *CDH1* may be prognostic indicators in LC.

## INTRODUCTION

Laryngeal cancer (LC) is a common type of malignant head and neck tumor, and the incidence is increasing yearly [1]. However, the etiology of LC remains unclear and the prognosis is poor. LC can result from both environmental and genetic factors [2, 3]. While the majority of LC patients have a history of smoking and alcohol consumption [4], only a small percentage of individuals with similar histories eventually develop LC. This suggests that genetic susceptibility underlies LC [5].

Host genetic factors may influence the prognosis of cancer patients. Recently, various genetic polymorphisms were associated with a risk of LC [6–9]. Polymorphisms may contribute to cancer susceptibility, progression, and response to therapy. Previous studies have primarily assessed associations between single nucleotide polymorphisms (SNPs) and LC risk using case-control models. Rare host genetic factors that influence the prognosis of advanced LC patients have been reported. Long-term longitudinal studies are required to evaluate the impact of SNPs on disease progression, treatment response, and patient survival.

In this study, we investigated 37 SNPs in 27 genes that were previously associated with head and neck cancers to determine whether they were associated with the prognosis of LC patients.

## RESULTS

The demographic and clinical characteristics of the LC patients are shown in Table 1. The median age of the patients was 60 years (range, 32–82). All of the patients were men who were metastasis-free. The mean follow-up period was 38 months (range: 3–122). There were 100 deaths at the time of the last observation. Overall, the median survival time was 48 months.

**Table 1:** Characteristics of patients included in this study

Variables	*N* (%)	*p* value
Total number of patients enrolled	170	
Age		> 0.05
< 60	80 (47.06)	
≥ 60	90 (52.94)	
Tumor differentiation		> 0.05
Well	31 (18.24)	
Moderate	125 (73.53)	
Poor	14 (8.23)	
pT		< 0.001
T1	40 (23.53)	
T2	62 (36.47)	
T3	50 (29.41)	
T4	18 (10.59)	
pN		< 0.001
N0	116 (68.23)	
N1	30 (17.65)	
N2	24 (14.12)	
WHO grade		< 0.001
I	37 (21.76)	
II	36 (21.18)	
III	61 (35.88)	
IV	36 (21.18)	
Surgery method		< 0.001
Partial laryngectomy	104 (61.18)	
Total laryngectomy	66 (38.82)	
Cervical lymph node dissection		> 0.05
Yes	37 (21.76)	
No	133 (78.24)	
No. patients with follow-up information available	170	
Median follow-up time, months	38	
Median survival time, months	48	
Status at last observation		
Alive	70 (41.18)	
Death	100 (58.82)	

T, pathologic tumor stage; N, pathologic nodal stage.

**Figure 1:**
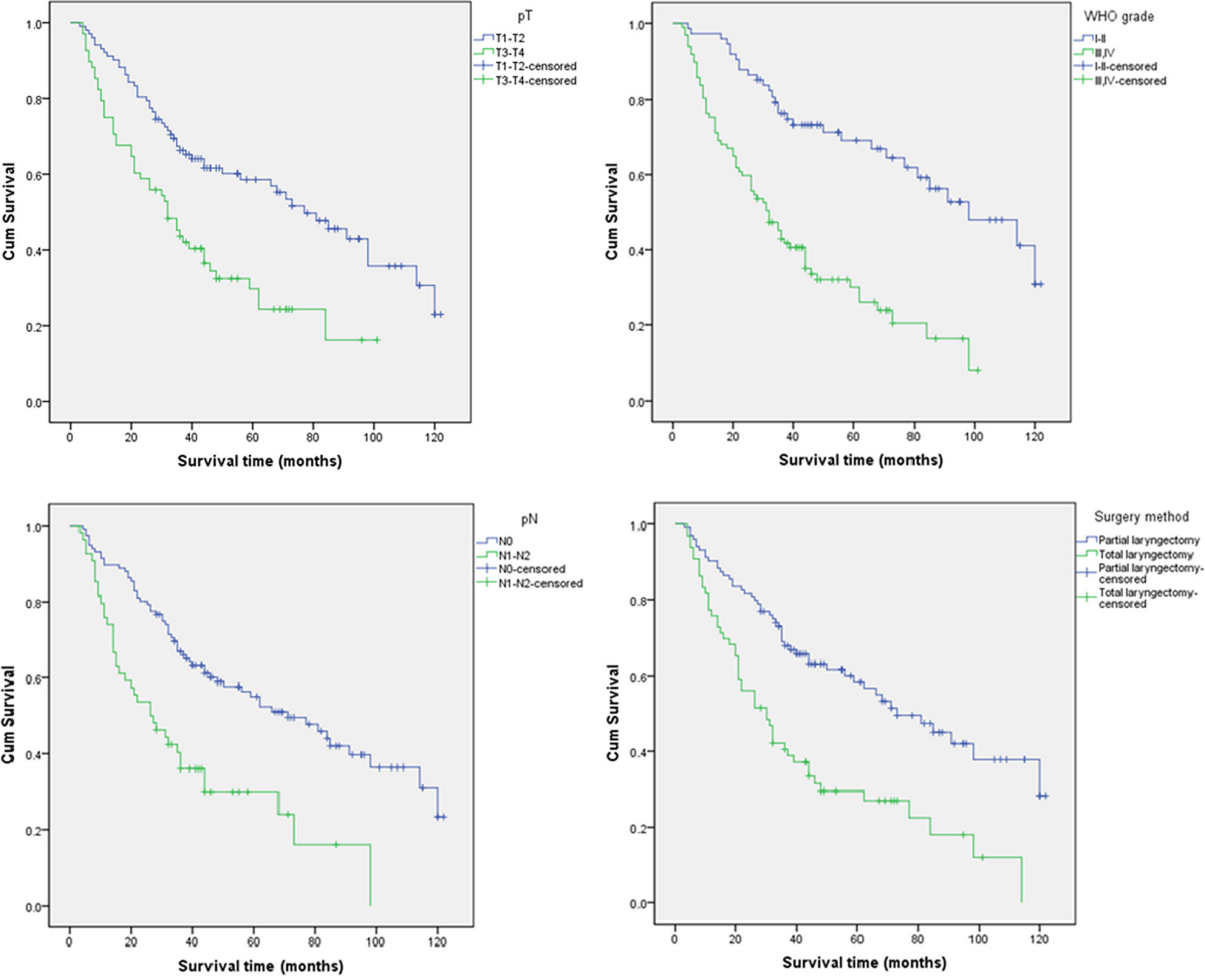
Kaplan-Meier analysis of LC patient overall survival according to the pT, pN, WHO grade, and surgical method

Clinical factors including age, pT, pN, WHO grade, degree of tumor differentiation, surgical method, and whether the patient underwent cervical lymph node dissection were assessed in a univariate analysis (Table 2). The distribution of the studied SNPs in the WHO grade was listed in Supplementary Table S1. We identified significant associations between clinical factors including the degree of tumor differentiation, pT, pN, WHO grade, and surgical method and LC patient prognosis. All of these factors increased the risk of mortality. Compared to patients with T1–T2 stage disease, N0, I–II grade, and who underwent partial laryngectomy, patients with T3–T4 stage, N1–N2, III–IV grade, and who underwent total laryngectomy had elevated risks of death, with HRs and 95% CIs of 2.17 (1.448–3.253), 2.394 (1.582–3.623), 3.298 (2.100–5.180) and 2.346 (1.576–3.492), respectively (Figure 1).

**Table 2:** Univariate analysis of the impact of clinical factors on prognosis for LC patients

Variables	Overall survival			
	Event/total	MST (months)	HR (95% CI)	Log-rank *p*
Age				
< 60	47/80	59.0	Ref	
≥ 60	53/90	48.0	1.161 (0.782–1.722)	0.456
Tumor differentiation				
Well	18/31	71.0	Ref	
Moderate	70/125	59.0	0.933 (0.556–1.567)	0.008
Poor	12/14	15.0	2.397 (1.150–4.997)	
pT				
T1–T2	52/102	77.0	Ref	
T3–T4	48/68	32.0	2.170 (1.448–3.253)	< 0.001
pN				
N0	61/116	71.0	Ref	
N1-N2	39/54	26.0	2.394 (1.582–3.623)	< 0.001
WHO grade				
I–II	30/73	98.0	Ref	
III–IV	70/97	32.0	3.298 (2.100–5.180)	< 0.001
Surgery method				
Partial laryngectomy	50/104	73.0	Ref	
Total laryngectomy	50/66	30.0	2.346 (1.576–3.492)	< 0.001
Cervical lymph node dissection				
Yes	19/37	36.0	Ref	
No	81/133	56.0	0.711 (0.425–1.189)	0.188

T, pathologic tumor stage; N, pathologic nodal stage;

MST, median survival time; HR, hazard ratio; 95% CI, 95% confidence interval.

**Figure 2:**
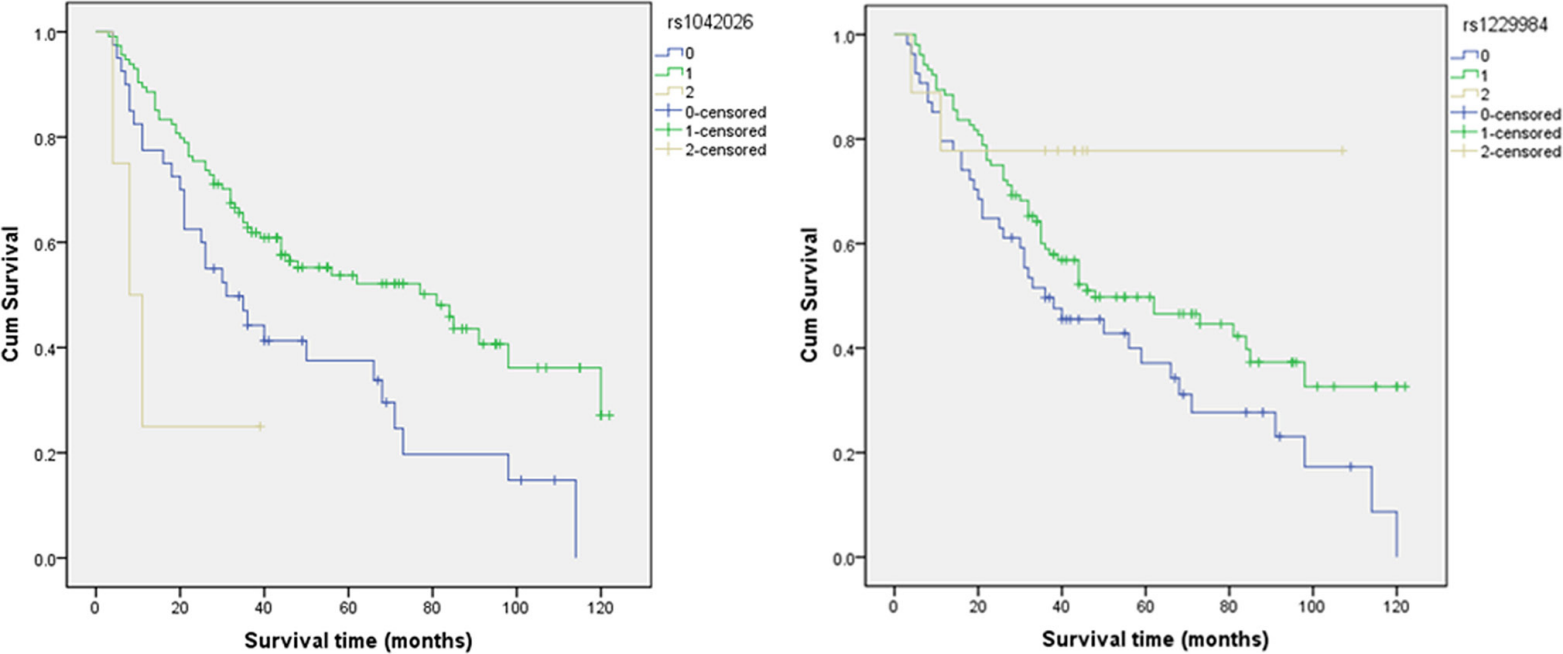
The individual effects of rs1042026 and rs1229984 on overall survival

The basic characteristics of all candidate SNPs that were analyzed in the study, including chromosome, position, band, alleles A/B, gene(s), and role(s), are shown in Table 3. Two of the 37 candidate SNPs evaluated showed statistically significantly correlations with overall survival (Table 4) according to Log-rank tests and Cox regression analysis. The A/G genotype of *alcohol dehydrogenase 1B (ADH1B)* rs1042026 (HR, 0.538; 95% CI, 0.345–0.839) and G/A genotype of rs1229984 (HR, 0.659; 95% CI, 0.438–0.991) were associated with increased overall survival. Kaplan-Meier curves of overall survival for the different genotypes of rs1042026 and rs1229984 are shown in Figure 2.

**Table 3:** Candidate SNP data

SNP ID	Chr	Position	Band	Alleles A/B	Gene(s)	Role
rs13130787	4	94887031	4q22.2	T/C		
rs3805322	4	100056998	4q23	G/A	*ADH4*	Intron
rs1042026	4	100228466	4q23	A/G	*ADH1B*	3′ UTR
rs1229984	4	100239319	4q23	G/A	*ADH1B*	Coding exon
rs1789924	4	100274286	4q23	T/C	*ADH1C*	Promoter
rs971074	4	100341861	4q23	A/G	*ADH7*	Coding exon
rs1000589	13	64141913	13q21.31	G/T		
rs1585440	13	66481815	13q21.32	A/C		
rs9573163	13	73908846	13q22.1	C/G		
rs9543325	13	73916628	13q22.1	T/C		
rs1886449	13	73932114	13q22.1	T/C		
rs2039553	13	80299722	13q31.1	G/A		
rs944289	14	36649246	14q13.3	T/C		
rs4444235	14	54410919	14q22.2	C/T	*BMP4*	Downstream
rs4779584	15	32994756	15q13.3	C/T	*SCG5*	Downstream
rs4785204	16	50103734	16q12.1	T/C	*HEATR3*	Intron
rs9929218	16	68820946	16q22.1	A/G	*CDH1*	Intron
rs17761864	17	2171637	17p13.3	A/C	*SMG6*	Intron
rs4924935	17	18753870	17p11.2	C/T	*PRPSAP2*	Promoter
rs225190	17	30877658	17q11.2	G/A	*MYO1D*	Intron
rs6503659	17	39897264	17q21.2	A/T	*HAP1*	Promoter
rs2257205	17	56448297	17q22	A/G	*RNF43*	Coding exon
rs2847281	18	12821593	18p11.21	C/T	*PTPN2*	Intron
rs12456874	18	13366862	18p11.21	G/A	*C18orf1*	Intron
rs4939827	18	46453463	18q21.1	T/C	*SMAD7*	Intron
rs7504990	18	50517776	18q21.2	T/C	*DCC*	Intron
rs961253	20	6404281	20p12.3	A/C		
rs2423279	20	7812350	20p12.3	C/T		
rs4925386	20	60921044	20q13.33	T/C	*LAMA5*	Intron (boundary)
rs372883	21	30717737	21q21.3	G/A	*BACH1*	Intron
rs455804	21	31146169	21q21.3	T/G	*NCRNA00110*	Downstream
rs2014300	21	36357861	21q22.12	A/G	*RUNX1*	Intron
rs1547374	21	43778895	21q22.3	G/A	*TFF1*	Downstream
rs4822983	22	29115066	22q12.1	T/C	*CHEK2*	Intron
rs738722	22	29130012	22q12.1	T/C	*HSCB*	Promoter
rs2239815	22	29192670	22q12.1	T/C	*XBP1*	Intron
rs5768709	22	48929569	22q13.32	A/G	*FAM19A5*	Intron

A/B, minor/major alleles; Chr, chromosome.

**Table 4:** Univariate analysis of the associations between the candidate SNPs and LC patient survival

SNP ID	Genotype	Event/total	MST (months)	HR (95% CI)	Log-rank *p*
rs13130787					
	C/C	19/38	98	Ref	
	T/C	71/113	39	1.638 (0.986–2.722)	0.152
	T/T	10/19	62	1.414 (0.656–3.049)	
rs3805322					
	A/A	22/39	73	Ref	
	G/A	59/98	44	0.982 (0.601–1.604)	0.997
	G/G	19/33	50	0.981 (0.529–1.818)	
rs1042026					
	G/G	30/40	31	Ref	
	A/G	58/114	81	0.538 (0.345–0.839)	0.001
	A/A	3/4	8	2.344 (0.709–7.741)	
rs1229984					
	A/A	39/54	36	Ref	
	G/A	57/104	48	0.659 (0.438–0.991)	0.043
	G/G	2/9		0.291 (0.070-1.206)	
rs1789924					
	C/C	95/158	46	Ref	
	T/C	4/11	84	0.542 (0.198–1.481)	0.222
	T/T				
rs971074					
	G/G	73/125	50	Ref	
	A/G	26/44	46	1.118 (0.714–1.751)	0.624
	A/A				
rs1000589					
	T/T	42/66	38	Ref	
	G/T	38/77	68	0.691 (0.444–1.075)	0.003
	G/G	20/26	22	1.711 (0.997-2.937)	
rs1585440					
	C/C	23/33	62	Ref	
	A/C	75/134	48	0.803 (0.502–1.286)	0.595
	A/A	1/2	11	1.299 (0.174–9.683)	
rs9573163					
	G/G	30/59	62	Ref	
	C/G	56/92	40	1.397 (0.896–2.180)	0.113
	C/C	14/19	36	1.882 (0.996–3.558)	
rs9543325					
	C/C	33/47	35	Ref	
	T/C	47/86	66	0.691 (0.443–1.080)	0.200
	T/T	20/37	59	0.674 (0.386–1.176)	
rs1886449					
	C/C	6/11	77	Ref	
	T/C	75/131	48	1.193 (0.518–2.746)	0.898
	T/T	2/2	20	1.365 (0.266–6.991)	
rs2039553					
	A/A	32/58	81	Ref	
	G/A	19/32	32	1.474 (0.832–2.612)	0.389
	G/G	19/36	62	1.075 (0.607–1.902)	
rs944289					
	C/C	16/26	62	Ref	
	T/C	79/137	50	0.712 (0.414–1.223)	0.068
	T/T	2/3	6	2.792 (0.638–12.221)	
rs4444235					
	T/T	25/34	40	Ref	
	C/T	63/115	59	0.762 (0.479–1.211)	0.506
	C/C	6/11	84	0.859 (0.352–2.098)	
rs4779584					
	T/T	65/103	48	Ref	
	C/T	30/57	62	0.805 (0.521–1.242)	0.611
	C/C	5/10	44	0.905 (0.363–2.256)	
rs4785204					
	C/C	51/87	46	Ref	
	T/C	32/54	59	1.002 (0.643–1.560)	0.803
	T/T	10/16	37	1.248 (0.631–2.467)	
rs9929218					
	G/G	70/118	48	Ref	
	A/G	27/48	73	0.855 (0.548–1.334)	0.081
	A/A	2/2	14	3.931 (0.946–16.324)	
rs17761864					
	C/C	75/120	46	Ref	
	A/C	16/37		0.660 (0.384–1.133)	0.087
	A/A	3/4	7	2.282 (0.714–7.297)	
rs4924935					
	T/T	80/130	44	Ref	
	C/T	13/28	98	0.596 (0.331–1.074)	0.110
	C/C	5/7	32	1.578 (0.635–3.920)	
rs225190					
	A/A	54/96	62	Ref	
	G/A	40/65	44	1.137 (0.754–1.715)	0.634
	G/G	4/6	18	1.528 (0.551–4.238)	
rs6503659					
	T/T	73/123	56	Ref	
	A/T	23/42	36	0.981 (0.614–1.569)	0.634
	A/A	2/3	22	1.935 (0.473–7.918)	
rs2257205					
	G/G	26/41	44	Ref	
	A/G	55/102	62	0.647 (0.403–1.039)	0.152
	A/A	16/24	48	0.891 (0.477–1.664)	
rs2847281					
	T/T	75/129	56	Ref	
	C/T	24/38	44	1.095 (0.690–1.739)	0.698
	C/C				
rs12456874					
	A/A	91/158	48	Ref	
	G/A	9/12	26	1.327 (0.667–2.638)	0.415
	G/G				
rs4939827					
	C/C	72/118	48	Ref	
	T/C	23/46	91	0.920 (0.575–1.473)	0.772
	T/T	1/1	36	1.818 (0.251–13.145)	
rs7504990					
	C/C	60/99	44	Ref	
	T/C	36/62	62	0.889 (0.587–1.347)	0.729
	T/T	4/9	71	0.720 (0.261–1.982)	
rs961253					
	C/C	87/146	44	Ref	
	A/C	13/24	68	0.928 (0.518–1.663)	0.801
	A/A				
rs2423279					
	T/T	65/117	48	Ref	
	C/T	30/47	59	0.976 (0.632–1.508)	0.912
	C/C	0/1			
rs4925386					
	C/C	59/107	62	Ref	
	T/C	17/25	40	1.160 (0.676–1.991)	0.608
	T/T	2/5		0.574 (0.139–2.370)	
rs372883					
	A/A	7/15		Ref	
	G/A	82/137	56	0.993 (0.457–2.159)	0.958
	G/G	1/2	11	1.331 (0.163–10.863)	
rs455804					
	G/G	47/84	62	Ref	
	T/G	42/69	44	1.228 (0.809–1.863)	0.126
	T/T	8/12	22	2.115 (0.989–4.520)	
rs2014300					
	G/G	73/127	48	Ref	0.522
	A/G	25/38	39	1.113 (0.706–1.755)	
	A/A	1/1	26	2.793 (0.385–20.276)	
rs1547374					
	A/A	22/38	56	Ref	
	G/A	61/106	59	0.994 (0.610–1.623)	0.330
	G/G	16/24	35	1.493 (0.777–2.866)	
rs4822983					
	C/C	66/114	50	Ref	
	T/C	29/45	44	1.051 (0.678–1.628)	0.976
	T/T	3/5	32	1.008 (0.312–3.257)	
rs738722					
	C/C	17/27	66	Ref	
	T/C	65/119	59	0.713 (0.417–1.218)	0.370
	T/T	3/4	5	1.113 (0.315–3.933)	
rs2239815					
	C/C	34/65	56	Ref	
	T/C	50/77	44	1.300 (0.840–2.012)	0.325
	T/T	6/11	114	1.113 (0.332–1.915)	
rs5768709					
	G/G	18/31	59	Ref	
	A/G	77/132	48	1.025 (0.613–1.715)	0.951
	A/A	5/7	35	1.173 (0.430–3.200)	

MST, median survival time; HR, hazard ratio; 95% CI, 95% confidence interval;

Long-rank *p* values were calculated using the Chi-Square test.

After adjusting for the various clinical factors, multivariate Cox regression analysis demonstrated that SNP genotype was an independent prognostic factor for overall survival. We identified significant correlations between two SNPs (*ADH1B* rs1229984 and *CDH1* rs9929218) and the prognosis of LC patients (Table 5). The G/A genotype of *ADH1B* rs1229984 was associated with increased overall survival (HR, 0.537; 95% CI, 0.340–0.848; *p* = 0.008), and the A/A genotype of *CDH1* rs9929218 with reduced overall survival (HR, 6.074; 95% CI, 1.426–25.870; *p* = 0.0015).

**Table 5:** Multivariate analysis of the associations between candidate SNPs and LC patient survival

SNP ID	Genotype	HR (95% CI)	*p*
rs13130787			
	C/C	Ref	
	T/C	1.641 (0.975–2.760)	0.062
	T/T	1.868 (0.856–4.073)	0.116
rs1042026			
	G/G	Ref	
	A/G	0.668 (0.420–1.060)	0.087
	A/A	1.898 (0.550–6.543)	0.310
rs1229984			
	A/A	Ref	
	G/A	0.537 (0.340–0.848)	0.008
	G/G	0.352 (0.084–1.477)	0.154
rs1000589			
	T/T	Ref	
	G/T	0.773 (0.483–1.236)	0.282
	G/G	1.734 (0.999–3.010)	0.050
rs9573163			
	G/G	Ref	
	C/G	1.515 (0.958–2.394)	0.075
	C/C	1.573 (0.811–3.050)	0.180
rs9543325			
	C/C	Ref	
	T/C	0.791 (0.505–1.240)	0.307
	T/T	0.673 (0.378–1.200)	0.180
rs944289			
	C/C	Ref	
	T/C	0.831 (0.477–1.448)	0.514
	T/T	2.391 (0.513–11.139)	0.267
rs9929218			
	G/G	Ref	
	A/G	0.998 (0.637–1.565)	0.994
	A/A	6.074 (1.426–25.870)	0.015
rs17761864			
	C/C	Ref	
	A/C	0.577 (0.328–1.018)	0.058
	A/A	1.82 (0.523–6.338)	0.347
rs4924935			
	T/T	Ref	
	C/T	0.692 (0.380–1.261)	0.229
	C/C	1.334 (0.501–3.549)	0.564
rs2257205			
	G/G	Ref	
	A/G	0.783 (0.478–1.284)	0.333
	A/A	0.863 (0.454–1.642)	0.654
rs455804			
	G/G	Ref	
	T/G	1.199 (0.783–1.838)	0.404
	T/T	1.872 (0.812–4.312)	0.141

HR, hazard ratio; 95% CI, 95% confidence interval.

All *p* values were calculated using the Wald test.

A *p* < 0.05 indicates statistical significance.

## DISCUSSION

We evaluated the effects of 37 SNPs in 26 genes on the prognosis of 170 Han Chinese male LC patients. We demonstrated that *ADH1B* rs1042026, *ADH1B* rs1229984, and *CDH1* rs9929218 were significantly associated with overall survival. Our data shed new light on the association between genetic variations in the *ADH1B* and *CDH1* genes and LC prognosis in the Han Chinese population.

The *ADH1B* gene is located on chromosome 4q21-q23. The rs1229984 variant in the *ADH1B* gene causes a missense mutation (R48H) which increases the activity of the *ADH1B* enzyme (i.e. faster acetaldehyde production generated by ethanol oxidation) [10, 11]. Following alcohol consumption, elevated ADH1B activity is thought to transiently increase the level of acetaldehyde, which leads to unpleasant effects that limit the desire to continue drinking. A meta-analysis of this variant in Asian, European, and African Americans populations (where the rs1229984 A allele is common) demonstrated a strong association with alcohol-related disorder risk [12–14].

The *CDH1* gene encodes the E-cadherin protein, which is a 120 kDa glycoprotein that consists of an extracellular domain containing five tandem repeats, a cytoplasmic domain, and a single transmembrane domain [15, 16]. *CDH1* hypermethylation is one of the mechanisms by which E-cadherin expression is silenced. Abnormal CDH1 expression has been linked to many human diseases including cancer, nephrolithiasis, pre-eclampsia, and ectopic pregnancy [17, 18]. Association between rs9929218 and both colorectal cancer risk and survival have also been observed [19–21]. Our results demonstrated that *CDH1* rs9929218 AA genotype was associated with reduced overall survival in the Chinese Han population. However, additional studies with larger cohorts derived from other populations are necessary.

Differences in survival were most apparent in individuals with T stage. There are several possible explanations for this finding. First, because individuals with T stage have already acquired many somatic mutations that could drive tumor growth or therapeutic resistance, subtle variations that alter the DNA repair capacity will not have a significant impact. Second, the differences in survival may reflect radiation-related outcomes, given that most T stage individuals received radiation treatment for the primary tumor, whereas only a minority of T stage individuals received radiation for treatment of the primary tumor. However, the latter explanation does not account for the common occurrence of relapsed metastatic disease outside the field of radiation.

In summary, our data raise the possibility that three polymorphisms may be one of major driving forces of LC progression, and could be valuable prognostic markers for LC patients. Further studies will focus on the functional experiments based on the relevant genes on animal models, to investigate detailed mechanism involved.

## MATERIALS AND METHODS

### Study participants

A total of 170 male patients (median age 60 years, range 32–82) who were diagnosed with LC at the First Affiliated Hospital of the Medical College of Xi'an Jiaotong University before 2002 and who were followed-up from January 2002 to April 2013 were included in the study. All patients underwent resection for LC at the same hospital. Additionally, all patients were Han Chinese from Xi'an city and the surrounding regions. The research protocol was performed according to the Declaration of Helsinki and was approved by the Human Research Committee of the First Affiliated Hospital of the Medical College of Xi'an Jiaotong University for the Approval of Research Involving Human Subjects. Informed consent was obtained from all patients.

### Demographic and clinical data

Patient demographic and clinical data including age, sex, ethnicity, residential region, smoking status, alcohol use, education status, body mass index, and family history of cancer were collected through in-person interviews using a standardized epidemiological questionnaire. Detailed clinical information including the time of diagnosis, time of surgery and/or treatment with chemotherapy, time of recurrence and/or death, tumor stage, degree of differentiation, location, whether lymph node dissection was performed, and the treatment protocol was collected through medical chart review or physician consultation. Standard follow-up was performed by a trained specialist through on-site interviews, direct calls, or written communication with either patients or family members. The most recent follow-up data in this analysis were obtained in April 2013. No patients were lost during follow-up.

### SNP selection and genotyping

We selected 37 SNPs with a minor allele frequency (MAF) > 5% in the HapMap Han Chinese population in Beijing that were previously associated with head and neck cancer [22–24] for genotyping. Genomic DNA was extracted from peripheral blood leukocytes using GoldMag^®^ nanoparticles (GoldMag Ltd. Xi'an, China) according to the manufacturer's instructions. DNA concentrations were estimated using a NanoDrop 2000 (Thermo Scientific, Waltham, Massachusetts, USA). The Sequenom MassARRAY Assay Design 3.0 Software was used to design Multiplexed SNP MassEXTEND assays [25]. Genotyping was performed using a Sequenom MassARRAY RS1000 [25]. Data management and analysis were performed using the Sequenom Typer 4.0 software as previously described [25, 26].

### Data analysis

All follow-up survey and experimental data were analyzed using SPSS 17.0 (SPSS, Chicago, IL, USA). Survival time was defined as the time between the date of diagnosis and either the date of death (deceased patients) or last contact date (living patients). The Kaplan-Meier method was used to estimate overall survival. The survival curves were compared using Log-rank tests. Univariate analysis included the following factors: age, degree of tumor differentiation, pathologic tumor stage (pT), pathologic nodal stage (pN), WHO grade, surgical method, whether cervical lymph node dissection was performed, and the 37 candidate SNPs. Univariate and multivariable Cox proportional hazard models were used to calculate hazard ratio (HRs) and 95% confidence intervals (CIs). Two-sided *p* values < 0.05 were considered statistically significant and were calculated using the Wald test.

## SUPPLEMENTARY MATERIALS TABLE




