# Supplementary material for: ADH1B and CDH1 polymorphisms predict prognosis in male patients with non-metastatic laryngeal cancer
Source: Oncotarget. 2016 Sep 28;7(45):73216–28. doi: 10.18632/oncotarget.12301 (PMC5341974; doi:10.18632/oncotarget.12301)
Supplement: Supplementary file 1 [file oncotarget-07-73216-s001.pdf]

ADH1B and *CDH1* polymorphisms predict prognosis in male patients with non-metastatic laryngeal cancer

Supplementary Materials

Supplementary Table S1: The distribution of the studied SNPs in the WHO grade. See Supplementary _Table_S1.
