# Supplementary material for: ADH1B and CDH1 polymorphisms predict prognosis in male patients with non-metastatic laryngeal cancer
Source: Oncotarget. 2016 Sep 28;7(45):73216–28. doi: 10.18632/oncotarget.12301 (PMC5341974; doi:10.18632/oncotarget.12301)
Supplement: Supplementary file 2 [file oncotarget-07-73216-s002.docx]

The distribution of the studied SNPs in the WHO grade

rs13130787

| WHO grade | | | Frequency | Percent | Valid Percent |
| --- | --- | --- | --- | --- | --- |
| Ⅰ | Valid | CC | 11 | 29.7 | 29.7 |
|  |  | CT | 21 | 56.8 | 56.8 |
|  |  | TT | 5 | 13.5 | 13.5 |
|  |  | Total | 37 | 100.0 | 100.0 |
| Ⅱ | Valid | CC | 7 | 19.4 | 19.4 |
|  |  | CT | 23 | 63.9 | 63.9 |
|  |  | TT | 6 | 16.7 | 16.7 |
|  |  | Total | 36 | 100.0 | 100.0 |
| Ⅲ | Valid | CC | 14 | 23.0 | 23.0 |
|  |  | CT | 44 | 72.1 | 72.1 |
|  |  | TT | 3 | 4.9 | 4.9 |
|  |  | Total | 61 | 100.0 | 100.0 |
| Ⅳ | Valid | CC | 6 | 16.7 | 16.7 |
|  |  | CT | 25 | 69.4 | 69.4 |
|  |  | TT | 5 | 13.9 | 13.9 |
|  |  | Total | 36 | 100.0 | 100.0 |

rs3805322

| WHO grade | | | Frequency | Percent | Valid Percent |
| --- | --- | --- | --- | --- | --- |
| Ⅰ | Valid | AA | 8 | 21.6 | 21.6 |
|  |  | AG | 21 | 56.8 | 56.8 |
|  |  | GG | 8 | 21.6 | 21.6 |
|  |  | Total | 37 | 100.0 | 100.0 |
| Ⅱ | Valid | AA | 11 | 30.6 | 30.6 |
|  |  | AG | 16 | 44.4 | 44.4 |
|  |  | GG | 9 | 25.0 | 25.0 |
|  |  | Total | 36 | 100.0 | 100.0 |
| Ⅲ | Valid | AA | 12 | 19.7 | 19.7 |
|  |  | AG | 42 | 68.9 | 68.9 |
|  |  | GG | 7 | 11.5 | 11.5 |
|  |  | Total | 61 | 100.0 | 100.0 |
| Ⅳ | Valid | AA | 8 | 22.2 | 22.2 |
|  |  | AG | 19 | 52.8 | 52.8 |
|  |  | GG | 9 | 25.0 | 25.0 |
|  |  | Total | 36 | 100.0 | 100.0 |

rs1042026

| WHO grade | | | Frequency | Percent | Valid Percent |
| --- | --- | --- | --- | --- | --- |
| Ⅰ | Valid | AG | 31 | 83.8 | 83.8 |
|  |  | GG | 6 | 16.2 | 16.2 |
|  |  | Total | 37 | 100.0 | 100.0 |
| Ⅱ | Valid | AG | 24 | 66.7 | 72.7 |
|  |  | GG | 9 | 25.0 | 27.3 |
|  |  | Total | 33 | 91.7 | 100.0 |
|  | Missing |  | 3 | 8.3 |  |
|  | Total | | 36 | 100.0 |  |
| Ⅲ | Valid | AA | 2 | 3.3 | 3.7 |
|  |  | AG | 38 | 62.3 | 70.4 |
|  |  | GG | 14 | 23.0 | 25.9 |
|  |  | Total | 54 | 88.5 | 100.0 |
|  | Missing |  | 7 | 11.5 |  |
|  | Total | | 61 | 100.0 |  |
| Ⅳ | Valid | AA | 2 | 5.6 | 5.9 |
|  |  | AG | 21 | 58.3 | 61.8 |
|  |  | GG | 11 | 30.6 | 32.4 |
|  |  | Total | 34 | 94.4 | 100.0 |
|  | Missing |  | 2 | 5.6 |  |
|  | Total | | 36 | 100.0 |  |

rs1229984

| WHO grade | | | Frequency | Percent | Valid Percent |
| --- | --- | --- | --- | --- | --- |
| Ⅰ | Valid | AA | 13 | 35.1 | 35.1 |
|  |  | GA | 21 | 56.8 | 56.8 |
|  |  | GG | 3 | 8.1 | 8.1 |
|  |  | Total | 37 | 100.0 | 100.0 |
| Ⅱ | Valid | AA | 14 | 38.9 | 41.2 |
|  |  | GA | 18 | 50.0 | 52.9 |
|  |  | GG | 2 | 5.6 | 5.9 |
|  |  | Total | 34 | 94.4 | 100.0 |
|  | Missing |  | 2 | 5.6 |  |
|  | Total | | 36 | 100.0 |  |
| Ⅲ | Valid | AA | 14 | 23.0 | 23.3 |
|  |  | GA | 45 | 73.8 | 75.0 |
|  |  | GG | 1 | 1.6 | 1.7 |
|  |  | Total | 60 | 98.4 | 100.0 |
|  | Missing |  | 1 | 1.6 |  |
|  | Total | | 61 | 100.0 |  |
| Ⅳ | Valid | AA | 13 | 36.1 | 36.1 |
|  |  | GA | 20 | 55.6 | 55.6 |
|  |  | GG | 3 | 8.3 | 8.3 |
|  |  | Total | 36 | 100.0 | 100.0 |

rs1789924

| WHO grade | | | Frequency | Percent | Valid Percent |
| --- | --- | --- | --- | --- | --- |
| Ⅰ | Valid | CC | 34 | 91.9 | 91.9 |
|  |  | CT | 3 | 8.1 | 8.1 |
|  |  | Total | 37 | 100.0 | 100.0 |
| Ⅱ | Valid | CC | 33 | 91.7 | 91.7 |
|  |  | CT | 3 | 8.3 | 8.3 |
|  |  | Total | 36 | 100.0 | 100.0 |
| Ⅲ | Valid | CC | 58 | 95.1 | 95.1 |
|  |  | CT | 3 | 4.9 | 4.9 |
|  |  | Total | 61 | 100.0 | 100.0 |
| Ⅳ | Valid | CC | 33 | 91.7 | 94.3 |
|  |  | CT | 2 | 5.6 | 5.7 |
|  |  | Total | 35 | 97.2 | 100.0 |
|  | Missing |  | 1 | 2.8 |  |
|  | Total | | 36 | 100.0 |  |

rs971074

| WHO grade | | | Frequency | Percent | Valid Percent |
| --- | --- | --- | --- | --- | --- |
| Ⅰ | Valid | AG | 6 | 16.2 | 16.2 |
|  |  | GG | 31 | 83.8 | 83.8 |
|  |  | Total | 37 | 100.0 | 100.0 |
| Ⅱ | Valid | AG | 6 | 16.7 | 16.7 |
|  |  | GG | 30 | 83.3 | 83.3 |
|  |  | Total | 36 | 100.0 | 100.0 |
| Ⅲ | Valid | AG | 21 | 34.4 | 34.4 |
|  |  | GG | 40 | 65.6 | 65.6 |
|  |  | Total | 61 | 100.0 | 100.0 |
| Ⅳ | Valid | AG | 11 | 30.6 | 31.4 |
|  |  | GG | 24 | 66.7 | 68.6 |
|  |  | Total | 35 | 97.2 | 100.0 |
|  | Missing |  | 1 | 2.8 |  |
|  | Total | | 36 | 100.0 |  |

rs1000589

| WHO grade | | | Frequency | Percent | Valid Percent |
| --- | --- | --- | --- | --- | --- |
| Ⅰ | Valid | GG | 3 | 8.1 | 8.1 |
|  |  | GT | 23 | 62.2 | 62.2 |
|  |  | TT | 11 | 29.7 | 29.7 |
|  |  | Total | 37 | 100.0 | 100.0 |
| Ⅱ | Valid | GG | 4 | 11.1 | 11.1 |
|  |  | GT | 19 | 52.8 | 52.8 |
|  |  | TT | 13 | 36.1 | 36.1 |
|  |  | Total | 36 | 100.0 | 100.0 |
| Ⅲ | Valid | GG | 11 | 18.0 | 18.3 |
|  |  | GT | 26 | 42.6 | 43.3 |
|  |  | TT | 23 | 37.7 | 38.3 |
|  |  | Total | 60 | 98.4 | 100.0 |
|  | Missing |  | 1 | 1.6 |  |
|  | Total | | 61 | 100.0 |  |
| Ⅳ | Valid | GG | 8 | 22.2 | 22.2 |
|  |  | GT | 9 | 25.0 | 25.0 |
|  |  | TT | 19 | 52.8 | 52.8 |
|  |  | Total | 36 | 100.0 | 100.0 |

rs1585440

| WHO grade | | | Frequency | Percent | Valid Percent |
| --- | --- | --- | --- | --- | --- |
| Ⅰ | Valid | AA | 1 | 2.7 | 2.7 |
|  |  | CA | 32 | 86.5 | 86.5 |
|  |  | CC | 4 | 10.8 | 10.8 |
|  |  | Total | 37 | 100.0 | 100.0 |
| Ⅱ | Valid | CA | 28 | 77.8 | 77.8 |
|  |  | CC | 8 | 22.2 | 22.2 |
|  |  | Total | 36 | 100.0 | 100.0 |
| Ⅲ | Valid | CA | 48 | 78.7 | 80.0 |
|  |  | CC | 12 | 19.7 | 20.0 |
|  |  | Total | 60 | 98.4 | 100.0 |
|  | Missing |  | 1 | 1.6 |  |
|  | Total | | 61 | 100.0 |  |
| Ⅳ | Valid | AA | 1 | 2.8 | 2.8 |
|  |  | CA | 26 | 72.2 | 72.2 |
|  |  | CC | 9 | 25.0 | 25.0 |
|  |  | Total | 36 | 100.0 | 100.0 |

rs9573163

| WHO grade | | | Frequency | Percent | Valid Percent |
| --- | --- | --- | --- | --- | --- |
| Ⅰ | Valid | CC | 1 | 2.7 | 2.7 |
|  |  | GC | 20 | 54.1 | 54.1 |
|  |  | GG | 16 | 43.2 | 43.2 |
|  |  | Total | 37 | 100.0 | 100.0 |
| Ⅱ | Valid | CC | 3 | 8.3 | 8.3 |
|  |  | GC | 22 | 61.1 | 61.1 |
|  |  | GG | 11 | 30.6 | 30.6 |
|  |  | Total | 36 | 100.0 | 100.0 |
| Ⅲ | Valid | CC | 7 | 11.5 | 11.5 |
|  |  | GC | 32 | 52.5 | 52.5 |
|  |  | GG | 22 | 36.1 | 36.1 |
|  |  | Total | 61 | 100.0 | 100.0 |
| Ⅳ | Valid | CC | 8 | 22.2 | 22.2 |
|  |  | GC | 18 | 50.0 | 50.0 |
|  |  | GG | 10 | 27.8 | 27.8 |
|  |  | Total | 36 | 100.0 | 100.0 |

rs9543325

| WHO grade | | | Frequency | Percent | Valid Percent |
| --- | --- | --- | --- | --- | --- |
| Ⅰ | Valid | CC | 9 | 24.3 | 24.3 |
|  |  | CT | 17 | 45.9 | 45.9 |
|  |  | TT | 11 | 29.7 | 29.7 |
|  |  | Total | 37 | 100.0 | 100.0 |
| Ⅱ | Valid | CC | 7 | 19.4 | 19.4 |
|  |  | CT | 24 | 66.7 | 66.7 |
|  |  | TT | 5 | 13.9 | 13.9 |
|  |  | Total | 36 | 100.0 | 100.0 |
| Ⅲ | Valid | CC | 20 | 32.8 | 32.8 |
|  |  | CT | 27 | 44.3 | 44.3 |
|  |  | TT | 14 | 23.0 | 23.0 |
|  |  | Total | 61 | 100.0 | 100.0 |
| Ⅳ | Valid | CC | 11 | 30.6 | 30.6 |
|  |  | CT | 18 | 50.0 | 50.0 |
|  |  | TT | 7 | 19.4 | 19.4 |
|  |  | Total | 36 | 100.0 | 100.0 |

rs1886449

| WHO grade | | | Frequency | Percent | Valid Percent |
| --- | --- | --- | --- | --- | --- |
| Ⅰ | Valid | CC | 2 | 5.4 | 5.9 |
|  |  | TC | 31 | 83.8 | 91.2 |
|  |  | TT | 1 | 2.7 | 2.9 |
|  |  | Total | 34 | 91.9 | 100.0 |
|  | Missing |  | 3 | 8.1 |  |
|  | Total | | 37 | 100.0 |  |
| Ⅱ | Valid | CC | 3 | 8.3 | 11.1 |
|  |  | TC | 24 | 66.7 | 88.9 |
|  |  | Total | 27 | 75.0 | 100.0 |
|  | Missing |  | 9 | 25.0 |  |
|  | Total | | 36 | 100.0 |  |
| Ⅲ | Valid | CC | 4 | 6.6 | 7.5 |
|  |  | TC | 48 | 78.7 | 90.6 |
|  |  | TT | 1 | 1.6 | 1.9 |
|  |  | Total | 53 | 86.9 | 100.0 |
|  | Missing |  | 8 | 13.1 |  |
|  | Total | | 61 | 100.0 |  |
| Ⅳ | Valid | CC | 2 | 5.6 | 6.7 |
|  |  | TC | 28 | 77.8 | 93.3 |
|  |  | Total | 30 | 83.3 | 100.0 |
|  | Missing |  | 6 | 16.7 |  |
|  | Total | | 36 | 100.0 |  |

rs2039553

| WHO grade | | | Frequency | Percent | Valid Percent |
| --- | --- | --- | --- | --- | --- |
| Ⅰ | Valid | AA | 15 | 40.5 | 53.6 |
|  |  | AG | 6 | 16.2 | 21.4 |
|  |  | GG | 7 | 18.9 | 25.0 |
|  |  | Total | 28 | 75.7 | 100.0 |
|  | Missing |  | 9 | 24.3 |  |
|  | Total | | 37 | 100.0 |  |
| Ⅱ | Valid | AA | 12 | 33.3 | 50.0 |
|  |  | AG | 9 | 25.0 | 37.5 |
|  |  | GG | 3 | 8.3 | 12.5 |
|  |  | Total | 24 | 66.7 | 100.0 |
|  | Missing |  | 12 | 33.3 |  |
|  | Total | | 36 | 100.0 |  |
| Ⅲ | Valid | AA | 24 | 39.3 | 57.1 |
|  |  | AG | 5 | 8.2 | 11.9 |
|  |  | GG | 13 | 21.3 | 31.0 |
|  |  | Total | 42 | 68.9 | 100.0 |
|  | Missing |  | 19 | 31.1 |  |
|  | Total | | 61 | 100.0 |  |
| Ⅳ | Valid | AA | 7 | 19.4 | 21.9 |
|  |  | AG | 12 | 33.3 | 37.5 |
|  |  | GG | 13 | 36.1 | 40.6 |
|  |  | Total | 32 | 88.9 | 100.0 |
|  | Missing |  | 4 | 11.1 |  |
|  | Total | | 36 | 100.0 |  |

rs944289

| WHO grade | | | Frequency | Percent | Valid Percent |
| --- | --- | --- | --- | --- | --- |
| Ⅰ | Valid | CC | 6 | 16.2 | 16.2 |
|  |  | CT | 31 | 83.8 | 83.8 |
|  |  | Total | 37 | 100.0 | 100.0 |
| Ⅱ | Valid | CC | 4 | 11.1 | 11.4 |
|  |  | CT | 30 | 83.3 | 85.7 |
|  |  | TT | 1 | 2.8 | 2.9 |
|  |  | Total | 35 | 97.2 | 100.0 |
|  | Missing |  | 1 | 2.8 |  |
|  | Total | | 36 | 100.0 |  |
| Ⅲ | Valid | CC | 8 | 13.1 | 13.6 |
|  |  | CT | 50 | 82.0 | 84.7 |
|  |  | TT | 1 | 1.6 | 1.7 |
|  |  | Total | 59 | 96.7 | 100.0 |
|  | Missing |  | 2 | 3.3 |  |
|  | Total | | 61 | 100.0 |  |
| Ⅳ | Valid | CC | 8 | 22.2 | 22.9 |
|  |  | CT | 26 | 72.2 | 74.3 |
|  |  | TT | 1 | 2.8 | 2.9 |
|  |  | Total | 35 | 97.2 | 100.0 |
|  | Missing |  | 1 | 2.8 |  |
|  | Total | | 36 | 100.0 |  |

rs4444235

| WHO grade | | | Frequency | Percent | Valid Percent |
| --- | --- | --- | --- | --- | --- |
| Ⅰ | Valid | CC | 2 | 5.4 | 6.1 |
|  |  | CT | 29 | 78.4 | 87.9 |
|  |  | TT | 2 | 5.4 | 6.1 |
|  |  | Total | 33 | 89.2 | 100.0 |
|  | Missing |  | 4 | 10.8 |  |
|  | Total | | 37 | 100.0 |  |
| Ⅱ | Valid | CC | 3 | 8.3 | 8.8 |
|  |  | CT | 21 | 58.3 | 61.8 |
|  |  | TT | 10 | 27.8 | 29.4 |
|  |  | Total | 34 | 94.4 | 100.0 |
|  | Missing |  | 2 | 5.6 |  |
|  | Total | | 36 | 100.0 |  |
| Ⅲ | Valid | CC | 5 | 8.2 | 8.3 |
|  |  | CT | 39 | 63.9 | 65.0 |
|  |  | TT | 16 | 26.2 | 26.7 |
|  |  | Total | 60 | 98.4 | 100.0 |
|  | Missing |  | 1 | 1.6 |  |
|  | Total | | 61 | 100.0 |  |
| Ⅳ | Valid | CC | 1 | 2.8 | 3.0 |
|  |  | CT | 26 | 72.2 | 78.8 |
|  |  | TT | 6 | 16.7 | 18.2 |
|  |  | Total | 33 | 91.7 | 100.0 |
|  | Missing |  | 3 | 8.3 |  |
|  | Total | | 36 | 100.0 |  |

rs4779584

| WHO grade | | | Frequency | Percent | Valid Percent |
| --- | --- | --- | --- | --- | --- |
| Ⅰ | Valid | CT | 14 | 37.8 | 37.8 |
|  |  | TT | 23 | 62.2 | 62.2 |
|  |  | Total | 37 | 100.0 | 100.0 |
| Ⅱ | Valid | CC | 3 | 8.3 | 8.3 |
|  |  | CT | 10 | 27.8 | 27.8 |
|  |  | TT | 23 | 63.9 | 63.9 |
|  |  | Total | 36 | 100.0 | 100.0 |
| Ⅲ | Valid | CC | 3 | 4.9 | 4.9 |
|  |  | CT | 20 | 32.8 | 32.8 |
|  |  | TT | 38 | 62.3 | 62.3 |
|  |  | Total | 61 | 100.0 | 100.0 |
| Ⅳ | Valid | CC | 4 | 11.1 | 11.1 |
|  |  | CT | 13 | 36.1 | 36.1 |
|  |  | TT | 19 | 52.8 | 52.8 |
|  |  | Total | 36 | 100.0 | 100.0 |

rs4785204

| WHO grade | | | Frequency | Percent | Valid Percent |
| --- | --- | --- | --- | --- | --- |
| Ⅰ | Valid | CC | 19 | 51.4 | 57.6 |
|  |  | CT | 13 | 35.1 | 39.4 |
|  |  | TT | 1 | 2.7 | 3.0 |
|  |  | Total | 33 | 89.2 | 100.0 |
|  | Missing |  | 4 | 10.8 |  |
|  | Total | | 37 | 100.0 |  |
| Ⅱ | Valid | CC | 17 | 47.2 | 51.5 |
|  |  | CT | 13 | 36.1 | 39.4 |
|  |  | TT | 3 | 8.3 | 9.1 |
|  |  | Total | 33 | 91.7 | 100.0 |
|  | Missing |  | 3 | 8.3 |  |
|  | Total | | 36 | 100.0 |  |
| Ⅲ | Valid | CC | 31 | 50.8 | 54.4 |
|  |  | CT | 17 | 27.9 | 29.8 |
|  |  | TT | 9 | 14.8 | 15.8 |
|  |  | Total | 57 | 93.4 | 100.0 |
|  | Missing |  | 4 | 6.6 |  |
|  | Total | | 61 | 100.0 |  |
| Ⅳ | Valid | CC | 20 | 55.6 | 58.8 |
|  |  | CT | 11 | 30.6 | 32.4 |
|  |  | TT | 3 | 8.3 | 8.8 |
|  |  | Total | 34 | 94.4 | 100.0 |
|  | Missing |  | 2 | 5.6 |  |
|  | Total | | 36 | 100.0 |  |

rs9929218

| WHO grade | | | Frequency | Percent | Valid Percent |
| --- | --- | --- | --- | --- | --- |
| Ⅰ | Valid | GA | 14 | 37.8 | 37.8 |
|  |  | GG | 23 | 62.2 | 62.2 |
|  |  | Total | 37 | 100.0 | 100.0 |
| Ⅱ | Valid | AA | 1 | 2.8 | 2.8 |
|  |  | GA | 9 | 25.0 | 25.0 |
|  |  | GG | 26 | 72.2 | 72.2 |
|  |  | Total | 36 | 100.0 | 100.0 |
| Ⅲ | Valid | GA | 17 | 27.9 | 28.8 |
|  |  | GG | 42 | 68.9 | 71.2 |
|  |  | Total | 59 | 96.7 | 100.0 |
|  | Missing |  | 2 | 3.3 |  |
|  | Total | | 61 | 100.0 |  |
| Ⅳ | Valid | AA | 1 | 2.8 | 2.8 |
|  |  | GA | 8 | 22.2 | 22.2 |
|  |  | GG | 27 | 75.0 | 75.0 |
|  |  | Total | 36 | 100.0 | 100.0 |

rs17761864

| WHO grade | | | Frequency | Percent | Valid Percent |
| --- | --- | --- | --- | --- | --- |
| Ⅰ | Valid | CA | 12 | 32.4 | 34.3 |
|  |  | CC | 23 | 62.2 | 65.7 |
|  |  | Total | 35 | 94.6 | 100.0 |
|  | Missing |  | 2 | 5.4 |  |
|  | Total | | 37 | 100.0 |  |
| Ⅱ | Valid | CA | 6 | 16.7 | 17.6 |
|  |  | CC | 28 | 77.8 | 82.4 |
|  |  | Total | 34 | 94.4 | 100.0 |
|  | Missing |  | 2 | 5.6 |  |
|  | Total | | 36 | 100.0 |  |
| Ⅲ | Valid | AA | 2 | 3.3 | 3.4 |
|  |  | CA | 14 | 23.0 | 23.7 |
|  |  | CC | 43 | 70.5 | 72.9 |
|  |  | Total | 59 | 96.7 | 100.0 |
|  | Missing |  | 2 | 3.3 |  |
|  | Total | | 61 | 100.0 |  |
| Ⅳ | Valid | AA | 2 | 5.6 | 6.1 |
|  |  | CA | 5 | 13.9 | 15.2 |
|  |  | CC | 26 | 72.2 | 78.8 |
|  |  | Total | 33 | 91.7 | 100.0 |
|  | Missing |  | 3 | 8.3 |  |
|  | Total | | 36 | 100.0 |  |

rs4924935

| WHO grade | | | Frequency | Percent | Valid Percent |
| --- | --- | --- | --- | --- | --- |
| Ⅰ | Valid | CC | 1 | 2.7 | 2.8 |
|  |  | CT | 10 | 27.0 | 27.8 |
|  |  | TT | 25 | 67.6 | 69.4 |
|  |  | Total | 36 | 97.3 | 100.0 |
|  | Missing |  | 1 | 2.7 |  |
|  | Total | | 37 | 100.0 |  |
| Ⅱ | Valid | CT | 5 | 13.9 | 14.7 |
|  |  | TT | 29 | 80.6 | 85.3 |
|  |  | Total | 34 | 94.4 | 100.0 |
|  | Missing |  | 2 | 5.6 |  |
|  | Total | | 36 | 100.0 |  |
| Ⅲ | Valid | CC | 5 | 8.2 | 8.3 |
|  |  | CT | 9 | 14.8 | 15.0 |
|  |  | TT | 46 | 75.4 | 76.7 |
|  |  | Total | 60 | 98.4 | 100.0 |
|  | Missing |  | 1 | 1.6 |  |
|  | Total | | 61 | 100.0 |  |
| Ⅳ | Valid | CC | 1 | 2.8 | 2.9 |
|  |  | CT | 4 | 11.1 | 11.4 |
|  |  | TT | 30 | 83.3 | 85.7 |
|  |  | Total | 35 | 97.2 | 100.0 |
|  | Missing |  | 1 | 2.8 |  |
|  | Total | | 36 | 100.0 |  |

rs225190

| WHO grade | | | Frequency | Percent | Valid Percent |
| --- | --- | --- | --- | --- | --- |
| Ⅰ | Valid | AA | 20 | 54.1 | 54.1 |
|  |  | AG | 16 | 43.2 | 43.2 |
|  |  | GG | 1 | 2.7 | 2.7 |
|  |  | Total | 37 | 100.0 | 100.0 |
| Ⅱ | Valid | AA | 23 | 63.9 | 65.7 |
|  |  | AG | 10 | 27.8 | 28.6 |
|  |  | GG | 2 | 5.6 | 5.7 |
|  |  | Total | 35 | 97.2 | 100.0 |
|  | Missing |  | 1 | 2.8 |  |
|  | Total | | 36 | 100.0 |  |
| Ⅲ | Valid | AA | 33 | 54.1 | 54.1 |
|  |  | AG | 27 | 44.3 | 44.3 |
|  |  | GG | 1 | 1.6 | 1.6 |
|  |  | Total | 61 | 100.0 | 100.0 |
| Ⅳ | Valid | AA | 20 | 55.6 | 58.8 |
|  |  | AG | 12 | 33.3 | 35.3 |
|  |  | GG | 2 | 5.6 | 5.9 |
|  |  | Total | 34 | 94.4 | 100.0 |
|  | Missing |  | 2 | 5.6 |  |
|  | Total | | 36 | 100.0 |  |

rs6503659

| WHO grade | | | Frequency | Percent | Valid Percent |
| --- | --- | --- | --- | --- | --- |
| Ⅰ | Valid | TA | 10 | 27.0 | 27.0 |
|  |  | TT | 27 | 73.0 | 73.0 |
|  |  | Total | 37 | 100.0 | 100.0 |
| Ⅱ | Valid | AA | 1 | 2.8 | 2.8 |
|  |  | TA | 5 | 13.9 | 13.9 |
|  |  | TT | 30 | 83.3 | 83.3 |
|  |  | Total | 36 | 100.0 | 100.0 |
| Ⅲ | Valid | AA | 2 | 3.3 | 3.4 |
|  |  | TA | 16 | 26.2 | 27.1 |
|  |  | TT | 41 | 67.2 | 69.5 |
|  |  | Total | 59 | 96.7 | 100.0 |
|  | Missing |  | 2 | 3.3 |  |
|  | Total | | 61 | 100.0 |  |
| Ⅳ | Valid | TA | 11 | 30.6 | 30.6 |
|  |  | TT | 25 | 69.4 | 69.4 |
|  |  | Total | 36 | 100.0 | 100.0 |

rs2257205

| WHO grade | | | Frequency | Percent | Valid Percent |
| --- | --- | --- | --- | --- | --- |
| Ⅰ | Valid | AA | 4 | 10.8 | 11.1 |
|  |  | AG | 28 | 75.7 | 77.8 |
|  |  | GG | 4 | 10.8 | 11.1 |
|  |  | Total | 36 | 97.3 | 100.0 |
|  | Missing |  | 1 | 2.7 |  |
|  | Total | | 37 | 100.0 |  |
| Ⅱ | Valid | AA | 4 | 11.1 | 11.4 |
|  |  | AG | 22 | 61.1 | 62.9 |
|  |  | GG | 9 | 25.0 | 25.7 |
|  |  | Total | 35 | 97.2 | 100.0 |
|  | Missing |  | 1 | 2.8 |  |
|  | Total | | 36 | 100.0 |  |
| Ⅲ | Valid | AA | 12 | 19.7 | 19.7 |
|  |  | AG | 31 | 50.8 | 50.8 |
|  |  | GG | 18 | 29.5 | 29.5 |
|  |  | Total | 61 | 100.0 | 100.0 |
| Ⅳ | Valid | AA | 4 | 11.1 | 11.4 |
|  |  | AG | 21 | 58.3 | 60.0 |
|  |  | GG | 10 | 27.8 | 28.6 |
|  |  | Total | 35 | 97.2 | 100.0 |
|  | Missing |  | 1 | 2.8 |  |
|  | Total | | 36 | 100.0 |  |

rs2847281

| WHO grade | | | Frequency | Percent | Valid Percent |
| --- | --- | --- | --- | --- | --- |
| Ⅰ | Valid | CT | 6 | 16.2 | 17.1 |
|  |  | TT | 29 | 78.4 | 82.9 |
|  |  | Total | 35 | 94.6 | 100.0 |
|  | Missing |  | 2 | 5.4 |  |
|  | Total | | 37 | 100.0 |  |
| Ⅱ | Valid | CT | 12 | 33.3 | 33.3 |
|  |  | TT | 24 | 66.7 | 66.7 |
|  |  | Total | 36 | 100.0 | 100.0 |
| Ⅲ | Valid | CT | 9 | 14.8 | 15.0 |
|  |  | TT | 51 | 83.6 | 85.0 |
|  |  | Total | 60 | 98.4 | 100.0 |
|  | Missing |  | 1 | 1.6 |  |
|  | Total | | 61 | 100.0 |  |
| Ⅳ | Valid | CT | 11 | 30.6 | 30.6 |
|  |  | TT | 25 | 69.4 | 69.4 |
|  |  | Total | 36 | 100.0 | 100.0 |

rs12456874

| WHO grade | | | Frequency | Percent | Valid Percent |
| --- | --- | --- | --- | --- | --- |
| Ⅰ | Valid | AA | 34 | 91.9 | 91.9 |
|  |  | AG | 3 | 8.1 | 8.1 |
|  |  | Total | 37 | 100.0 | 100.0 |
| Ⅱ | Valid | AA | 32 | 88.9 | 88.9 |
|  |  | AG | 4 | 11.1 | 11.1 |
|  |  | Total | 36 | 100.0 | 100.0 |
| Ⅲ | Valid | AA | 57 | 93.4 | 93.4 |
|  |  | AG | 4 | 6.6 | 6.6 |
|  |  | Total | 61 | 100.0 | 100.0 |
| Ⅳ | Valid | AA | 35 | 97.2 | 97.2 |
|  |  | AG | 1 | 2.8 | 2.8 |
|  |  | Total | 36 | 100.0 | 100.0 |

rs4939827

| WHO grade | | | Frequency | Percent | Valid Percent |
| --- | --- | --- | --- | --- | --- |
| Ⅰ | Valid | CC | 25 | 67.6 | 69.4 |
|  |  | CT | 11 | 29.7 | 30.6 |
|  |  | Total | 36 | 97.3 | 100.0 |
|  | Missing |  | 1 | 2.7 |  |
|  | Total | | 37 | 100.0 |  |
| Ⅱ | Valid | CC | 25 | 69.4 | 69.4 |
|  |  | CT | 11 | 30.6 | 30.6 |
|  |  | Total | 36 | 100.0 | 100.0 |
| Ⅲ | Valid | CC | 44 | 72.1 | 77.2 |
|  |  | CT | 12 | 19.7 | 21.1 |
|  |  | TT | 1 | 1.6 | 1.8 |
|  |  | Total | 57 | 93.4 | 100.0 |
|  | Missing |  | 4 | 6.6 |  |
|  | Total | | 61 | 100.0 |  |
| Ⅳ | Valid | CC | 24 | 66.7 | 66.7 |
|  |  | CT | 12 | 33.3 | 33.3 |
|  |  | Total | 36 | 100.0 | 100.0 |

rs7504990

| WHO grade | | | Frequency | Percent | Valid Percent |
| --- | --- | --- | --- | --- | --- |
| Ⅰ | Valid | CC | 21 | 56.8 | 56.8 |
|  |  | TC | 14 | 37.8 | 37.8 |
|  |  | TT | 2 | 5.4 | 5.4 |
|  |  | Total | 37 | 100.0 | 100.0 |
| Ⅱ | Valid | CC | 17 | 47.2 | 47.2 |
|  |  | TC | 15 | 41.7 | 41.7 |
|  |  | TT | 4 | 11.1 | 11.1 |
|  |  | Total | 36 | 100.0 | 100.0 |
| Ⅲ | Valid | CC | 39 | 63.9 | 63.9 |
|  |  | TC | 21 | 34.4 | 34.4 |
|  |  | TT | 1 | 1.6 | 1.6 |
|  |  | Total | 61 | 100.0 | 100.0 |
| Ⅳ | Valid | CC | 22 | 61.1 | 61.1 |
|  |  | TC | 12 | 33.3 | 33.3 |
|  |  | TT | 2 | 5.6 | 5.6 |
|  |  | Total | 36 | 100.0 | 100.0 |

rs961253

| WHO grade | | | Frequency | Percent | Valid Percent |
| --- | --- | --- | --- | --- | --- |
| Ⅰ | Valid | CA | 7 | 18.9 | 18.9 |
|  |  | CC | 30 | 81.1 | 81.1 |
|  |  | Total | 37 | 100.0 | 100.0 |
| Ⅱ | Valid | CA | 3 | 8.3 | 8.3 |
|  |  | CC | 33 | 91.7 | 91.7 |
|  |  | Total | 36 | 100.0 | 100.0 |
| Ⅲ | Valid | CA | 9 | 14.8 | 14.8 |
|  |  | CC | 52 | 85.2 | 85.2 |
|  |  | Total | 61 | 100.0 | 100.0 |
| Ⅳ | Valid | CA | 5 | 13.9 | 13.9 |
|  |  | CC | 31 | 86.1 | 86.1 |
|  |  | Total | 36 | 100.0 | 100.0 |

rs2423279

| WHO grade | | | Frequency | Percent | Valid Percent |
| --- | --- | --- | --- | --- | --- |
| Ⅰ | Valid | CC | 1 | 2.7 | 2.7 |
|  |  | TC | 8 | 21.6 | 21.6 |
|  |  | TT | 28 | 75.7 | 75.7 |
|  |  | Total | 37 | 100.0 | 100.0 |
| Ⅱ | Valid | TC | 11 | 30.6 | 30.6 |
|  |  | TT | 25 | 69.4 | 69.4 |
|  |  | Total | 36 | 100.0 | 100.0 |
| Ⅲ | Valid | TC | 20 | 32.8 | 35.1 |
|  |  | TT | 37 | 60.7 | 64.9 |
|  |  | Total | 57 | 93.4 | 100.0 |
|  | Missing |  | 4 | 6.6 |  |
|  | Total | | 61 | 100.0 |  |
| Ⅳ | Valid | TC | 8 | 22.2 | 22.9 |
|  |  | TT | 27 | 75.0 | 77.1 |
|  |  | Total | 35 | 97.2 | 100.0 |
|  | Missing |  | 1 | 2.8 |  |
|  | Total | | 36 | 100.0 |  |

rs4925386

| WHO grade | | | Frequency | Percent | Valid Percent |
| --- | --- | --- | --- | --- | --- |
| Ⅰ | Valid | CC | 22 | 59.5 | 78.6 |
|  |  | CT | 3 | 8.1 | 10.7 |
|  |  | TT | 3 | 8.1 | 10.7 |
|  |  | Total | 28 | 75.7 | 100.0 |
|  | Missing |  | 9 | 24.3 |  |
|  | Total | | 37 | 100.0 |  |
| Ⅱ | Valid | CC | 23 | 63.9 | 82.1 |
|  |  | CT | 5 | 13.9 | 17.9 |
|  |  | Total | 28 | 77.8 | 100.0 |
|  | Missing |  | 8 | 22.2 |  |
|  | Total | | 36 | 100.0 |  |
| Ⅲ | Valid | CC | 36 | 59.0 | 73.5 |
|  |  | CT | 12 | 19.7 | 24.5 |
|  |  | TT | 1 | 1.6 | 2.0 |
|  |  | Total | 49 | 80.3 | 100.0 |
|  | Missing |  | 12 | 19.7 |  |
|  | Total | | 61 | 100.0 |  |
| Ⅳ | Valid | CC | 26 | 72.2 | 81.3 |
|  |  | CT | 5 | 13.9 | 15.6 |
|  |  | TT | 1 | 2.8 | 3.1 |
|  |  | Total | 32 | 88.9 | 100.0 |
|  | Missing |  | 4 | 11.1 |  |
|  | Total | | 36 | 100.0 |  |

rs372883

| WHO grade | | | Frequency | Percent | Valid Percent |
| --- | --- | --- | --- | --- | --- |
| Ⅰ | Valid | AA | 2 | 5.4 | 5.9 |
|  |  | AG | 32 | 86.5 | 94.1 |
|  |  | Total | 34 | 91.9 | 100.0 |
|  | Missing |  | 3 | 8.1 |  |
|  | Total | | 37 | 100.0 |  |
| Ⅱ | Valid | AA | 1 | 2.8 | 3.1 |
|  |  | AG | 31 | 86.1 | 96.9 |
|  |  | Total | 32 | 88.9 | 100.0 |
|  | Missing |  | 4 | 11.1 |  |
|  | Total | | 36 | 100.0 |  |
| Ⅲ | Valid | AA | 5 | 8.2 | 8.9 |
|  |  | AG | 51 | 83.6 | 91.1 |
|  |  | Total | 56 | 91.8 | 100.0 |
|  | Missing |  | 5 | 8.2 |  |
|  | Total | | 61 | 100.0 |  |
| Ⅳ | Valid | AA | 7 | 19.4 | 21.9 |
|  |  | AG | 23 | 63.9 | 71.9 |
|  |  | GG | 2 | 5.6 | 6.3 |
|  |  | Total | 32 | 88.9 | 100.0 |
|  | Missing |  | 4 | 11.1 |  |
|  | Total | | 36 | 100.0 |  |

rs455804

| WHO grade | | | Frequency | Percent | Valid Percent |
| --- | --- | --- | --- | --- | --- |
| Ⅰ | Valid | GG | 21 | 56.8 | 58.3 |
|  |  | GT | 15 | 40.5 | 41.7 |
|  |  | Total | 36 | 97.3 | 100.0 |
|  | Missing |  | 1 | 2.7 |  |
|  | Total | | 37 | 100.0 |  |
| Ⅱ | Valid | GG | 15 | 41.7 | 42.9 |
|  |  | GT | 18 | 50.0 | 51.4 |
|  |  | TT | 2 | 5.6 | 5.7 |
|  |  | Total | 35 | 97.2 | 100.0 |
|  | Missing |  | 1 | 2.8 |  |
|  | Total | | 36 | 100.0 |  |
| Ⅲ | Valid | GG | 34 | 55.7 | 55.7 |
|  |  | GT | 25 | 41.0 | 41.0 |
|  |  | TT | 2 | 3.3 | 3.3 |
|  |  | Total | 61 | 100.0 | 100.0 |
| Ⅳ | Valid | GG | 14 | 38.9 | 42.4 |
|  |  | GT | 11 | 30.6 | 33.3 |
|  |  | TT | 8 | 22.2 | 24.2 |
|  |  | Total | 33 | 91.7 | 100.0 |
|  | Missing |  | 3 | 8.3 |  |
|  | Total | | 36 | 100.0 |  |

rs2014300

| WHO grade | | | Frequency | Percent | Valid Percent |
| --- | --- | --- | --- | --- | --- |
| Ⅰ | Valid | AG | 6 | 16.2 | 17.1 |
|  |  | GG | 29 | 78.4 | 82.9 |
|  |  | Total | 35 | 94.6 | 100.0 |
|  | Missing |  | 2 | 5.4 |  |
|  | Total | | 37 | 100.0 |  |
| Ⅱ | Valid | AG | 10 | 27.8 | 27.8 |
|  |  | GG | 26 | 72.2 | 72.2 |
|  |  | Total | 36 | 100.0 | 100.0 |
| Ⅲ | Valid | AA | 1 | 1.6 | 1.7 |
|  |  | AG | 13 | 21.3 | 21.7 |
|  |  | GG | 46 | 75.4 | 76.7 |
|  |  | Total | 60 | 98.4 | 100.0 |
|  | Missing |  | 1 | 1.6 |  |
|  | Total | | 61 | 100.0 |  |
| Ⅳ | Valid | AG | 9 | 25.0 | 25.7 |
|  |  | GG | 26 | 72.2 | 74.3 |
|  |  | Total | 35 | 97.2 | 100.0 |
|  | Missing |  | 1 | 2.8 |  |
|  | Total | | 36 | 100.0 |  |

rs1547374

| WHO grade | | | Frequency | Percent | Valid Percent |
| --- | --- | --- | --- | --- | --- |
| Ⅰ | Valid | AA | 9 | 24.3 | 25.0 |
|  |  | GA | 25 | 67.6 | 69.4 |
|  |  | GG | 2 | 5.4 | 5.6 |
|  |  | Total | 36 | 97.3 | 100.0 |
|  | Missing |  | 1 | 2.7 |  |
|  | Total | | 37 | 100.0 |  |
| Ⅱ | Valid | AA | 8 | 22.2 | 22.2 |
|  |  | GA | 23 | 63.9 | 63.9 |
|  |  | GG | 5 | 13.9 | 13.9 |
|  |  | Total | 36 | 100.0 | 100.0 |
| Ⅲ | Valid | AA | 14 | 23.0 | 23.0 |
|  |  | GA | 37 | 60.7 | 60.7 |
|  |  | GG | 10 | 16.4 | 16.4 |
|  |  | Total | 61 | 100.0 | 100.0 |
| Ⅳ | Valid | AA | 7 | 19.4 | 20.0 |
|  |  | GA | 21 | 58.3 | 60.0 |
|  |  | GG | 7 | 19.4 | 20.0 |
|  |  | Total | 35 | 97.2 | 100.0 |
|  | Missing |  | 1 | 2.8 |  |
|  | Total | | 36 | 100.0 |  |

rs4822983

| WHO grade | | | Frequency | Percent | Valid Percent |
| --- | --- | --- | --- | --- | --- |
| Ⅰ | Valid | CC | 22 | 59.5 | 64.7 |
|  |  | CT | 11 | 29.7 | 32.4 |
|  |  | TT | 1 | 2.7 | 2.9 |
|  |  | Total | 34 | 91.9 | 100.0 |
|  | Missing |  | 3 | 8.1 |  |
|  | Total | | 37 | 100.0 |  |
| Ⅱ | Valid | CC | 26 | 72.2 | 72.2 |
|  |  | CT | 9 | 25.0 | 25.0 |
|  |  | TT | 1 | 2.8 | 2.8 |
|  |  | Total | 36 | 100.0 | 100.0 |
| Ⅲ | Valid | CC | 42 | 68.9 | 70.0 |
|  |  | CT | 15 | 24.6 | 25.0 |
|  |  | TT | 3 | 4.9 | 5.0 |
|  |  | Total | 60 | 98.4 | 100.0 |
|  | Missing |  | 1 | 1.6 |  |
|  | Total | | 61 | 100.0 |  |
| Ⅳ | Valid | CC | 24 | 66.7 | 70.6 |
|  |  | CT | 10 | 27.8 | 29.4 |
|  |  | Total | 34 | 94.4 | 100.0 |
|  | Missing |  | 2 | 5.6 |  |
|  | Total | | 36 | 100.0 |  |

rs738722

| WHO grade | | | Frequency | Percent | Valid Percent |
| --- | --- | --- | --- | --- | --- |
| Ⅰ | Valid | CC | 3 | 8.1 | 8.8 |
|  |  | TC | 30 | 81.1 | 88.2 |
|  |  | TT | 1 | 2.7 | 2.9 |
|  |  | Total | 34 | 91.9 | 100.0 |
|  | Missing |  | 3 | 8.1 |  |
|  | Total | | 37 | 100.0 |  |
| Ⅱ | Valid | CC | 8 | 22.2 | 28.6 |
|  |  | TC | 20 | 55.6 | 71.4 |
|  |  | Total | 28 | 77.8 | 100.0 |
|  | Missing |  | 8 | 22.2 |  |
|  | Total | | 36 | 100.0 |  |
| Ⅲ | Valid | CC | 7 | 11.5 | 13.0 |
|  |  | TC | 46 | 75.4 | 85.2 |
|  |  | TT | 1 | 1.6 | 1.9 |
|  |  | Total | 54 | 88.5 | 100.0 |
|  | Missing |  | 7 | 11.5 |  |
|  | Total | | 61 | 100.0 |  |
| Ⅳ | Valid | CC | 9 | 25.0 | 26.5 |
|  |  | TC | 23 | 63.9 | 67.6 |
|  |  | TT | 2 | 5.6 | 5.9 |
|  |  | Total | 34 | 94.4 | 100.0 |
|  | Missing |  | 2 | 5.6 |  |
|  | Total | | 36 | 100.0 |  |

rs2239815

| WHO grade | | | Frequency | Percent | Valid Percent |
| --- | --- | --- | --- | --- | --- |
| Ⅰ | Valid | CC | 16 | 43.2 | 48.5 |
|  |  | CT | 15 | 40.5 | 45.5 |
|  |  | TT | 2 | 5.4 | 6.1 |
|  |  | Total | 33 | 89.2 | 100.0 |
|  | Missing |  | 4 | 10.8 |  |
|  | Total | | 37 | 100.0 |  |
| Ⅱ | Valid | CC | 14 | 38.9 | 41.2 |
|  |  | CT | 17 | 47.2 | 50.0 |
|  |  | TT | 3 | 8.3 | 8.8 |
|  |  | Total | 34 | 94.4 | 100.0 |
|  | Missing |  | 2 | 5.6 |  |
|  | Total | | 36 | 100.0 |  |
| Ⅲ | Valid | CC | 17 | 27.9 | 31.5 |
|  |  | CT | 32 | 52.5 | 59.3 |
|  |  | TT | 5 | 8.2 | 9.3 |
|  |  | Total | 54 | 88.5 | 100.0 |
|  | Missing |  | 7 | 11.5 |  |
|  | Total | | 61 | 100.0 |  |
| Ⅳ | Valid | CC | 18 | 50.0 | 56.3 |
|  |  | CT | 13 | 36.1 | 40.6 |
|  |  | TT | 1 | 2.8 | 3.1 |
|  |  | Total | 32 | 88.9 | 100.0 |
|  | Missing |  | 4 | 11.1 |  |
|  | Total | | 36 | 100.0 |  |

rs5768709

| WHO grade | | | Frequency | Percent | Valid Percent |
| --- | --- | --- | --- | --- | --- |
| Ⅰ | Valid | AA | 1 | 2.7 | 2.7 |
|  |  | AG | 31 | 83.8 | 83.8 |
|  |  | GG | 5 | 13.5 | 13.5 |
|  |  | Total | 37 | 100.0 | 100.0 |
| Ⅱ | Valid | AA | 2 | 5.6 | 5.6 |
|  |  | AG | 26 | 72.2 | 72.2 |
|  |  | GG | 8 | 22.2 | 22.2 |
|  |  | Total | 36 | 100.0 | 100.0 |
| Ⅲ | Valid | AA | 2 | 3.3 | 3.3 |
|  |  | AG | 47 | 77.0 | 77.0 |
|  |  | GG | 12 | 19.7 | 19.7 |
|  |  | Total | 61 | 100.0 | 100.0 |
| Ⅳ | Valid | AA | 2 | 5.6 | 5.6 |
|  |  | AG | 28 | 77.8 | 77.8 |
|  |  | GG | 6 | 16.7 | 16.7 |
|  |  | Total | 36 | 100.0 | 100.0 |
